# Association between anemia and falls in community-dwelling older people: cross-sectional results from the KORA-Age study

**DOI:** 10.1186/1471-2318-14-29

**Published:** 2014-03-07

**Authors:** Kathrin Thaler-Kall, Angela Döring, Annette Peters, Barbara Thorand, Eva Grill, Wolfgang Koenig, Alexander Horsch, Christa Meisinger

**Affiliations:** 1 Institute of Epidemiology II, Helmholtz Zentrum München, German Research Center for Environmental Health, Neuherberg, Germany; 2 Institute for Medical Statistics and Epidemiology (IMSE), Technical University Munich (TUM), Munich, Germany; 3 Department of Medical Information Processing, Biometry and Epidemiology, and German Center for Vertigo and Balance Disorders, Ludwig-Maximilians Universität (LMU), Munich, Germany; 4 Department of Internal Medicine II-Cardiology, University of Ulm Medical Center, Ulm, Germany; 5 Department of Computer Science, MI&T Group, University of Tromsø, Tromsø, Norway; 6 Department of Clinical Medicine, Telemedicine and eHealth Group, University of Tromsø, Tromsø, Norway; 7 Central Hospital of Augsburg, MONICA/KORA Myocardial Infarction Registry, Augsburg, Germany

## Abstract

**Background:**

Falls and fractures are among the principal causes of disability, and mortality of older people. Therefore, identifying treatable risk factors for falls in this population is very important. Here we evaluate the association between anemia and falls in community-dwelling people aged 65 years and older.

**Methods:**

In 2009 967 community-dwelling people aged 65 years and older were included as part of the KORA-Age study. History of falls was assessed via questions derived from the National Health and Nutrition Examination Survey questionnaire. A non-fasting venous blood sample was obtained from all study participants. Anemia was defined as a hemoglobin level below 12 g/dL in women and below 13 g/dL in men according to the WHO criteria. Different logistic regression models were computed including relevant confounders such as sex, age, and disability to estimate Odds Ratios (OR) for falls.

**Results:**

In the total sample there was no significant association between anemia and falls neither in the unadjusted (OR 1.35; 95% CI 0.87-2.09) nor in the multivariable-adjusted models (OR 1.06; 95% CI 0.66-1.70). The association between continuous hemoglobin levels and falls was significant in the unadjusted model (OR per 1 SD decrease 1.36; 95% CI 1.14-1.64), but after adjustment for age and sex the association was attenuated and lost its significance (OR 1.13; 95% CI 0.92-1.38). In age- and sex-stratified analyses, no significant associations between anemia or hemoglobin levels and falls could be found. However, in joint analysis in the total sample a significantly, more than two-fold increased risk was observed after multivariable adjustment in persons with anemia and disability (OR 2.10; 95% CI 1.12-3.93) in comparison to persons without anemia and disability.

**Conclusions:**

In the present study we have not found an independent association between hemoglobin levels or anemia and falls in older people from the general population. Because there was an additive effect of anemia and disability on the occurrence of falls, blood count should be measured in disabled older men and women to identify persons, who are at particular high risk for falls.

## Background

Falls and fall-related injuries are considered a major public health problem in older people. Thirty percent of people aged 65 plus experience a fall at least once a year, and 15% at least twice per year [1-3]. It has been well established knowledge that falls and fractures are among the principal causes of disability, admission to hospital care, and mortality in older people [4-6]. Due to the inability to get up again without help, older people sometimes do experience a “long lie” [7], thus further aggravation of injuries occurs. It has been shown that half of older people who did lie longer after having fallen, die within 6 months [7,8]. Falls are also associated with high costs. For Germany, Heinrich et al. estimated overall attributable costs between 2.1 and 3.8 billion Euros per year [9]. Due to these serious health and economic consequences, the identification of treatable risk factors for falls of older people is important.

Anemia is common in old age, with a prevalence of approximately 11% in subjects aged 65 years and older as documented in the Third National Health and Nutrition Examination Survey (NHANES III) study. The prevalence of anemia increases rapidly after the age of 50, approaching a rate greater than 20% in those individuals aged 85 years or older [10]. This also has been confirmed by the Leiden 85 plus study, in which the prevalence of anemia has been 26.7% for people aged 85 years or older [11]. Regarding sex differences the results of the NHANES III study showed almost no difference in the group of 65 to 74 years (7.8% in men vs. 8.5% in women), yet with increasing age, the prevalence of anemia was much higher in men than in women (26.1% vs. 20.1%; aged 85+) [10]. In the Leiden 85 plus study the difference was even higher: 35.5% anemic males vs. 22.3% anemic females [11].

Recently, anemia in old age, defined as a hemoglobin level below 12 g/dL in women and below 13 g/dL in men [12], has received increasing attention among researchers. Symptoms of anemia include low energy, fatigue, dizziness, and general weakness. In addition, late life anemia is associated with subsequent physical decline, as illustrated by increased disability, impaired performance, and muscle weakness [13,14]. With these consequences, anemia could result in an increased risk of subsequent falls, although research confirming such a potential link is limited.

So far, only a few publications exist investigating the relation of anemia and falls in older persons. Three studies found a significant increase in risk of falls with decreasing hemoglobin (Hb) levels [15-17]. One other could not find a significant relation, but points out that anemic people fall more often than non-anemic people [18]. Two of these publications included only people living in nursing homes [15] or hospitalized for acute care [17], and two others included community-dwelling people with conflicting results [16,18].

Therefore, the aim of this study was to analyze the association between anemia and falls in 967 community-dwelling men and women aged 65 years or older. Furthermore, we examined the combined relationship of anemia and disability as well as frailty with falls in older people.

## Methods

### Study design and participants

Data were collected in 2009 during the KORA (Cooperative Health Research in the Region of Augsburg)-Age study, a follow-up study of the four MONICA/KORA Augsburg Surveys [19]. People aged 65 years and older were taken from a population based random sample (n = 5,991). In total, 4,127 people participated in a standardized telephone interview (response 67%). Out of this group a randomly drawn sample of 1,079 participants additionally underwent extensive physical examinations including the registration of drug intake, collection of blood samples, anthropometric examination, grip force measurement, gait analysis, and an additional interview amongst others. Blood samples and answers to fall questionnaires were available for 1,048 participants. After exclusion of 81 participants because of missing data the final data set for the present analysis consisted of 967 participants (477 women and 490 men) aged 65–91 years. The study was approved by the ethics committee of the “Bayerische Landesärztekammer”; all participants provided a written informed consent.

### Outcome definition

History of falls was assessed with the question “Did you fall in the previous year?” with possible answers “yes, once”, “yes, more than once” and “no”. This variable was dichotomized in “at least one fall” and “no fall”. The question was derived from the National Health and Nutrition Examination Survey (NHANES)-questionnaire [20].

A non-fasting venous blood sample was obtained from all study participants while sitting. Hematological parameters were determined from fresh venous EDTA blood samples using impedance measurements (Coulter® LH 780, Beckman Coulter GmbH, Krefeld, Germany). Anemia was defined according to WHO criteria as a hemoglobin (Hb)-level less than 12 g/dL in women and less than 13 g/dL in men [12].

### Covariables

Information on socio-demographic characteristics and lifestyle behavior was collected by an interview. Frailty was assessed according to the criteria of Fried et al. [21]: weight loss in the last 6 months (short German SCREEN II questionnaire [22]), fatigue (via interview questions), low physical activity (via interview questions [23]), low walking speed (Timed Up and Go Test) and low grip force (measured via JAMAR, Saehan Corp.). Participants were classified as frail when they met 3 or more criteria, pre-frail when they met 1 or 2 criteria and non-frail when no criterion was met. The variable was dichotomized in “frail” (including pre-frail) and “non-frail”. The disability score was assessed via the Stanford Health Assessment Questionnaire [24] and categorized as disability “yes” (score ≥0.5) or “no” (score <0.5) as suggested in literature [25]. Multimorbidity was obtained with the Charlson-Comorbidity-Index from Chaudhry [26]. Participants were classified as multimorbid, if they had ≥3 diseases. Drug intake during the last 7 days before the examination was recorded with the IDOM-Software [27].

### Statistical analyses

Besides descriptive analyses, different logistic regression models were computed to assess associations of anemia/hemoglobin values with falls, respectively. Hemoglobin levels were considered as continuous variable (decrease per SD) and anemia (yes/no) was defined via WHO criteria [12].

Variables were considered as confounders in the multivariable regression model, if they were significantly (p <0.05) related to falls as well as Hb-level/anemia after adjustment for age and sex (for details see Table 1). However, the number of drugs was considered as confounder although it was not significantly related to falls, because the relation to anemia and Hb-level was significant and literature suggests it as one risk factor for falls [28-30].

**Table 1:** Overview of p-values for considered confounders

	**Hb level**	**Anemic-WHO **[12]	**Falls**
Multimorbidity	0.0049	0.0118	0.1334
Disability	0.0023	<.0001	0.0038
Diuretics	0.0584	0.0318	0.6326
Frailty	<.0001	<.0001	0.1866
Hypertension	0.1297	0.8939	0.2585
Diabetes	0.0004	0.0369	0.4392
Anticoagulants	0.2342	0.0625	0.2856
Antihypertensives	0.1361	0.0553	0.9232
Number of drugs	<.0001	<.0001	0.3257
Nutrition score	0.7864	0.1241	0.0044
Elevated alcohol intake^*^	0.0004	0.0702	0.4226
PASE^†^	0.7922	0.2476	0.7308
BMI	0.0012	0.0689	0.0274

^*^alcohol intake >24 g/day in men and >12 g/day in women.

^†^Physical Activity Scale for the Elderly. Scores range from 0 to 361 [31].

For the outcome “falls (yes/no)” the unadjusted model included hemoglobin level or anemia as independent variables. Model 1 additionally included age (continuous), and sex (in the unstratified analyses only) as confounders. In model 2, the number of drugs was added in addition to the confounders of model 1. In model 3 the variable “disability status (yes/no)” was added to the confounders of model 2.

We stratified all models by sex, as the normal Hb-level for men (≥13.0 g/dL) is higher than for women (≥12.0 g/dL) and due to the differences regarding the frequency of falls among men and women. We also stratified all models by age groups. The group of “young-old” included participants aged 65 to 74 years; the group of the “old-old” included all participants aged 75 years and older. Linearity was checked by including a quadratic term of the hemoglobin variable in the models (total study sample). As the p-values of the quadratic terms were not significant, linearity was assumed for the parameter hemoglobin.

To examine the joint effect of anemia and disability as well as frailty on the occurrence of falls, in a second step combined anemia and disability and anemia and frailty variables were created. The subjects were classified into four categories: 1. anemia “yes” and disability (frailty) “yes”, 2. anemia “yes” and disability (frailty) “no”, and 3. anemia “no” and disability (frailty) “yes”. Those with no anemia and no disability (frailty) were category 4 and thus the reference group. These analyses were performed in the total sample but not conducted separately for men and women or for the two age-groups due to low numbers in the different subgroups.

Results of the models were presented as Odds Ratios (ORs) and corresponding 95% confidence intervals (CIs). Significance tests were two tailed and p-values ≤0.05 were considered as statistically significant. All analyses were performed with SAS (version 9.2, SAS Institute Inc, Cary, NC, USA).

## Results

### Study sample characteristics by sex

Table 2 presents the study characteristics of the study population by sex. A total of 967 participants were included accounting 477 women and 490 men. The mean Hb-level was significantly higher in males than in females (14.0 vs. 13.0 g/dL, p < .0001). Also mean hematocrit was higher in men than in women. Women fell nearly twice as often as men. When determining the anemia status via WHO criteria (Hb-level <12 g/dL in female, Hb-level <13 g/dL in male) [12], 17.7% of all participants were classified as anemic. The percentage was higher in men, but differed not significantly from women. Altogether 30.8% of the examined women were disabled; the corresponding number in men was 19.6%. Other significant differences between men and women could be found for nutrition score (39.0 vs. 37.5), percentage of elevated alcohol intake (30.2% vs. 24.5%) and for the mean PASE value (124.7 vs. 113.9).

**Table 2:** Study sample characteristics by sex (n (percentages) or means (±SD)

**Variable**	**Males (n = 490)**	**Females (n = 477)**	**All (n = 967)**	**P value**
Mean age, y	75.9 (6.4)	76.0 (6.6)	76.0 (6.5)	0.7831^*^
Falls, n (%)	52 (10.6%)	91 (19.1%)	143 (14.0%)	0.0002^†^
Anemia-WHO [12] n (%)	96 (19.6%)	75 (15.7%)	171 (17.7%)	0.1149^†^
Mean hemoglobin, g/dL	14.0 (1.3)	13.0 (1.0)	-	<.0001^*^
Mean hematocrit, %	41.0 (3.8)	38.3 (3.0)	-	<.0001^*^
Multimorbidity, n (%)	44 (9.0%)	38 (8.0%)	82 (8.5%)	0.5718^†^
Disability, n (%)	91 (18.6%)	147 (30.8%)	238 (24.6%)	<.0001^†^
Frailty, n (%)	205 (41.8%)	196 (41.1%)	401 (41.5%)	0.8137^†^
Hypertension, n (%)	367 (74.9%)	359 (75.3%)	726 (75.1%)	0.8959^†^
Diabetes, n (%)	87 (17.8%)	80 (16.8%)	167 (17.3%)	0.6858^†^
Use of diuretics, n (%)	226 (46.1%)	204 (42.8%)	430 (44.5%)	0.2939^†^
Use of anticoagulants, n (%)	40 (8.2%)	38 (8.0%)	78 (8.1%)	0.9105^†^
Use of antihypertensives, n (%)	336 (68.6%)	328 (68.8%)	664 (68.7%)	0.9488^†^
Use of ≥5 prescribed drugs, n (%)	156 (31.8%)	149 (31.2%)	305 (31.5%)	0.8409^†^
Mean nutrition score	39.0 (5.1)	37.5 (5.4)	38.3 (5.3)	<.0001^*^
Elevated alcohol intake, n (%)^**^	148 (30.2%)	117 (24.5%)	265 (27.4%)	0.0479^†^
Mean PASE^††^	124.7 (60.4)	113.9 (48.9)	119.4 (55.2)	0.0025^*^
Mean BMI kg/m^2^	28.4 (3.9)	28.4 (4.6)	28.4 (4.2)	0.9249^*^

^*^t-test.

^†^Chi-square test.

^**^alcohol intake >24 g/day in men and >12 g/day in women.

^††^Physical Activity Scale for the Elderly. Scores range from 0 to 361 [31].

### Fall and non-fall-participant characteristics

Table 3 documents the study characteristics for participants who fell (fallers) as well as participants who reported no fall during the last year (non-fallers). In the total sample fallers were significantly older than non-fallers; the same could be found when analyzing men and women separately. Fallers also had lower values of hemoglobin and hematocrit in the total sample, but not in the sex-stratified groups. The percentage of anemic persons in the whole sample was higher for fallers than for non-fallers (21.2 vs. 17.0%); this was also the case when stratifying by sex, but the differences were not statistically significant. Significantly more fallers were frail and disabled. The nutrition score was significantly lower in fallers compared to non-fallers in the total sample. When stratifying by sex, differences were still significant among women, but in men only the difference regarding the proportion of disabled persons was statistically significant. In the total sample fallers had a significantly lower BMI than non-fallers, but these differences were not significant in both men and women. In women, the percentage of multimorbidity was significantly higher among fallers than among non-fallers.

**Table 3:** Fall and non-fall-participant characteristics (percentages or means (±SD))

**Variable**	**Fall participants (n = 143)**			**Non fall participants (n = 824)**			**p-value (*t*-test/Chi-square test)**		
	**All**	**Male (n = 52)**	**Female (n = 91)**	**All**	**Male (n = 438)**	**Female (n = 386)**	**All**	**Male**	**Female**
**Mean Age, y**	78.3 (7.3)	78.0 (7.5)	78.4 (7.2)	75.6 (6.3)	75.7 (6.2)	75.5 (6.3)	<.0001	0.0368	0.0004
**Mean hemoglobin, g/dL**	13.2 (1.2)	13.7 (1.4)	12.9 (0.9)	13.6 (1.3)	14.0 (1.3)	13.1 (1.1)	0.0005	0.1592	0.0880
**Anemia,% **[12]	21.7	28.9	17.6	17.0	18.5	15.3	0.1750	0.0753	0.5881
**Mean hematocrit, %**	38.8 (3.4)	40.4 (3.9)	37.9 (2.7)	39.8 (3.7)	41.1 (3.7)	38.4 (3.1)	0.0014	0.2190	0.1529
**Multimorbidity, %**	12.6	11.5	13.2	7.8	8.7	6.7	0.0561	0.4948	0.0409
**Disability, %**	41.3	34.6	45.1	21.7	16.7	27.5	<.0001	0.0017	0.0011
**Frailty, %**	52.5	46.2	56.0	39.6	41.3	37.6	0.0039	0.5045	0.0013
**Hypertension %**	72.7	82.7	67.0	75.5	74.0	77.2	0.4815	0.1704	0.0431
**History of diabetes %**	16.1	19.3	14.3	17.5	17.6	17.4	0.6844	0.7684	0.4805
**Use of diuretics %**	49.7	53.9	47.3	43.6	45.2	41.7	0.1767	0.2373	0.3363
**Use of anticoagulants %**	11.9	11.5	12.1	7.4	7.8	7.0	0.0690	0.3471	0.1065
**Use of anti-hypertensives %**	72.0	76.9	69.2	68.1	67.6	68.7	0.3478	0.1700	0.9148
**Use of ≥5 prescribed drugs %**	37.8	36.5	38.5	30.5	31.3	29.5	0.0828	0.4414	0.0983
**Nutrition score**	36.6 (6.0)	38.3 (6.2)	35.7 (5.7)	38.6 (5.1)	39.1 (4.9)	38.0 (5.3)	<.0001	0.2502	0.0006
Increased alcohol intake %^*^	22.4	25.0	20.9	28.3	30.8	25.4	0.1443	0.3873	0.3684
**PASE**^†^	112.8 (53.5)	114.5 (57.4)	111.8 (51.5)	120.5 (55.5)	125.9 (60.7)	114.4 (48.3)	0.1150	0.1846	0.6584
**BMI kg/m**^**2**^	27.7 (3.9)	27.6 (4.1)	27.7 (3.9)	28.6 (4.3)	28.6 (3.8)	28.6 (4.7)	0.0129	0.1021	0.1027

^*^alcohol intake >24 g/day in men and >12 g/day in women.

^†^Physical Activity Scale for the Elderly. Scores range from 0 to 361 [31].

### Logistic regression analysis

In the logistic regression models no independent association between hemoglobin levels or anemia and falls could be found. Detailed results for the different logistic regression models are provided in Table 4. In the total sample there was a significant association between hemoglobin levels and falls only in the unadjusted model (OR per 1 SD decrease 1.36; 95% CI 1.14-1.64). After adjustment for age and sex, the association already lost significance. In age- and sex-stratified analyses also no independent association between hemoglobin levels and falls could be found.

**Table 4:** Results of logistic regression models

	**Unadjusted model**	**Model 1**	**Model 2**	**Model 3**
***Hemoglobin-level (continuous, per SD decrease)***				
Total sample	**1.36 (1.14-1.64)**	1.13 (0.92-1.38)	1.11 (0.90-1.37)	1.09 (0.89-1.34)
Age 65-74	1.25 (0.91-1.73)	1.12 (0.78-1.61)	1.09 (0.75-1.57)	1.10 (0.76-1.59)
Age >74	**1.35 (1.09-1.69)**	1.23 (0.97-1.57)	1.21 (0.94-1.55)	1.15 (0.89-1.48)
Men	1.25 (0.94-1.65)	1.15 (0.87-1.54)	1.14 (0.85-1.55)	1.11 (0.83-1.49)
Age 65-74	0.98 (0.60-1.62)		1.00 (0.60-1.66)	1.00 (0.60-1.66)
Age >74	1.33 (0.94-1.87)		1.29 (0.89-1.85)	1.19 (0.83-1.73)
Women	1.25 (0.95-1.64)	1.11 (0.83-1.47)	1.09 (0.81-1.45)	1.07 (0.80-1.43)
Age 65-74	1.28 (0.77-2.13)		1.21 (0.72-2.03)	1.22 (0.72-2.06)
Age >74	1.15 (0.83-1.61)		1.14 (0.81-1.60)	1.09 (0.76-1.54)
***Anemia definition according WHO*** [12]				
Total sample	1.35 (0.87-2.09)	1.18 (0.75-1.86)	1.13 (0.71-1.80)	1.06 (0.66-1.70)
Age 65-74	1.24 (0.50-3.12)	1.23 (0.49-3.09)	1.17 (0.46-2.97)	1.21 (0.47-3.09)
Age >74	1.24 (0.74-2.05)	1.35 (0.81-2.26)	1.29 (0.76-2.19)	1.12 (0.65-1.92)
Men	1.79 (0.94-3.41)	1.51 (0.78-2.95)	1.49 (0.75-2.97)	1.35 (0.67-2.71)
Age 65-74	1.13 (0.24-5.30)		1.18 (0.25-5.61)	1.18 (0.24-5.90)
Age >74	1.85 (0.88-3.91)		1.73 (0.79-3.78)	1.50 (0.68-3.32)
Women	1.18 (0.65-2.17)	0.96 (0.52-1.80)	0.92 (0.49-1.74)	0.87 (0.46-1.66)
Age 65-74	1.29 (0.41-4.09)		1.20 (0.37-3.85)	1.22 (0.38-3.94)
Age >74	1.03 (0.50-2.11)		1.00 (0.48-2.08)	0.86 (0.40-1.81)
***Joint analyses (total sample)***				
Anemic & frail	**1.82 (1.05-3.15)**	1.32 (0.74-2.38)	1.27 (0.70-2.30)	
Anemic & not frail	1.51 (0.73-3.14)	1.54 (0.73-3.25)	1.49 (0.70-3.16)	
Not anemic & frail	**1.76 (1.17-2.63)**	1.40 (0.91-2.15)	1.38 (0.90-2.13)	
Not anemic & not frail	1.00	1.00	1.00	
Anemic & disabled	**2.99 (1.69-5.29)**	**2.17 (1.18-3.99)**	**2.10 (1.12-3.93)**	
Anemic & not disabled	0.94 (0.48-1.84)	0.87 (0.44-1.72)	0.85 (0.43-1.70)	
Not anemic & disabled	**2.32 (1.50-3.58)**	**1.64 (1.03-2.61)**	**1.61 (1.00-2.58)**	
Not anemic & not disabled	1.00	1.00	1.00	

Model 1: adjusted for age (in years, only in the whole sample and in sex-stratified analyses) and sex (only in the whole sample and in the age-stratified analyses).

Model 2: in addition to model 1 adjusted for number of drugs.

Model 3: in addition to model 2 adjusted for disability.

Significant results are written in bold.

When examining the association between anemia and falls no significant relationship was found, either in the unadjusted or in the confounder-adjusted models (Table 4). This could be shown for the whole group as well for women and men and both age-groups (young-old and old-old) in the stratified analyses.

### Combined analyses

The joint relationships between anemia and disability as well as frailty for the total sample are also shown in Table 4. Men and women without anemia and without disability (frailty) were the reference group. Persons with anemia and disability showed the strongest association with falls in comparison to the reference group, even after adjustment for age, sex and number of drugs (OR 2.10; 95% CI 1.12-3.93). The corresponding OR was 1.61 (95% CI 1.00-2.58) in disabled participants without anemia; however, persons with anemia but without disability had no significantly increased OR for falls compared to the reference group. The joint associations of anemia and frailty showed also an increased odd of falls in the unadjusted analysis across the 4 categories with the highest odds in persons with anemia and frailty in comparison to the reference group. After further adjustment, these associations were attenuated and became non-significant (see Table 4).

## Discussion

In the present analysis including 967 community-dwelling elderly men and women no significant association between hemoglobin levels or anemia and falls could be found. Thus, the suggestion that anemia is an independent risk factor of falls in elderly people from the general population could not be supported by these results. However, we found an additive effect of anemia and disability on the occurrence of falls.

On the contrary to our study prior studies were mostly conducted in selected groups [15-17] in women only [32] or they used self-reported data regarding anemia status or information from chart reviews [33]. Furthermore, most of the former analyses were based on smaller study samples [15,17,18].

The group of Duh et al. employed a retrospective open-cohort design and analyzed data of 47,350 individuals regarding anemia and risk of injurious falls in community-dwelling elderly people. Results showed that anemia increased the risk of injurious falls by 1.47 times in multivariate analysis adjusting for age, gender, health plan, history of falls, co-morbidities, and concomitant medications [16]. A 50% higher risk of fall injuries in community-dwelling adults aged 65 and older was also reported by Herndon et al. [33]. However, contrary to the present study, in that case–control study (467 cases, 691 controls), anemia based on self-report was used. Both studies mentioned above concentrated on injurious falls whereas our database includes also falls which did not cause injuries.

Pandya et al. found even more than twice the risk of falling for anemic participants when retrospectively analyzing the relationship between anemia and falls in 564 nursing home residents. However, in that study, only a selection of relevant covariables was available and collected by chart review [15]. Dharmarajan et al. investigated in a case–control study the relationship between the presence of anemia and falls during hospitalization in 362 ambulatory adults aged 59–104 years from long-term care and community settings. They found a 22% decreased risk of falls for every 1.0 g/dL increase in Hb-concentration and a 1.9 times increased risk of falls in anemic hospitalized patients [17].

In another study, Lawlor et al. analyzed data of 4,050 female participants of the population-based British Women's Heart and Health Study aged 60 to 79 and found that women reporting at least one fall per year had lower mean Hb-concentrations than women who did not report a fall; the inverse association remained even after adjustment for social class, BMI, chronic diseases, and each class of drug used (OR of any falls for a 1 SD increase of Hb was 0.90, 95% CI 0.81-0.99) [32]. In contrast to these findings we could not find significantly lower Hb-concentrations for women who reported a fall. Additionally we found no significant relationship of Hb-concentrations and falls in women. Contrary to the other publications the observational study of Penninx et al. including 394 community-dwelling older persons from The Netherlands could not prove that anemia directly affects risk of falls although longitudinal data were used and disease and functioning status were adjusted for. The authors hypothesized that anemia could still be only a manifestation of disease severity which would be the true link between anemia and falls [18].

So far, no data are available showing the joint association of anemia and disability with the occurrence of falls in the elderly general population. The results of our study extend the understanding that different factors in elderly people can have an additive effect on the occurrence of falls. Although no independent association between anemia and falls was found in the present sample, it could be shown, that persons with disability and concomitant present anemia represent a high risk group for falls. The findings from the present data therefore suggest that in elderly, mainly multimorbid persons single risk factors should not be considered isolated in relation to certain outcomes, but rather in the context of other relevant disorders and restrictions.

We recognize that our study has limitations. First, participants were asked for falls which happened in the past year on the day the blood sample was taken. Therefore, the anemia status of a participant could have been different at the time when the fall occurred. However, it could be assumed that in the present sample almost all persons with anemia suffered from chronic and not from acute anemia and thus were well adapted to the low hemoglobin levels. Hence, it is most likely that the status of anemia was similar at both points of time. Second, the interview-based assessment of falls could be imprecise, as the interrogated person could have forgotten these events (consistency bias), or did not want to report them in order to not appear frail. We did not find an investigation on the reliability and validity of the NHANES questionnaire [20] used to assess falls, but the short and easy way to ask people about their history of falls indicates reliable results. Third, the exclusion of participants who could not come to the study center because of severe illnesses could have influenced the results as they would most likely have lower hemoglobin levels. This could possibly have led to an underestimation of the true association between anemia and falls. On the other hand it was necessary to exclude people who could not walk anymore as they would not experience falls. Fourth, the cross-sectional design of the study represents a limitation, implicating that cause and effect relationships cannot be discerned. Fifth we included the intermediary variable disability as confounder in the logistic regression model which could result in a decreased association between anemia and falls. But as the results of the logistic regression models were already not significant before including these variable, this should not have influenced our results.

Finally, another issue worth considering would be the number of falls, as it was analyzed by Penninx et al., who found a 1.91 times greater risk of recurrent falls in elderly people with anemia (n = 394) [18]. But in our study group only 92 participants fell once, and 51 fell more than once. When looking at the anemic participants only 22 people fell once and only 9 fell more than once. We considered these groups as too small for meaningful analyses. The strength of the study is the inclusion of a relative large number of individuals randomly drawn from the general population, and the availability of data on lifestyle, medication, and multiple metabolic risk factors.

## Conclusion

In this analysis an independent relationship of anemia and falls in older people from the general population could not be demonstrated. The relationship shown in other studies including hospitalized or nursing home living participants or focusing on injurious falls could not be confirmed by our study population. Because there was an additive effect of anemia and disability on the occurrence of falls, blood count should be measured in disabled older men and women to identify persons, who are at particular high risk for falls. Further studies, in particular prospective studies need to be carried out to clarify the role of anemia regarding the occurrence of falls in elderly people from the general population.

## Competing interests

The authors report no potential conflicts of interest.

## Authors’ contributions

KTK did the statistical analysis, generated all tables and drafted the manuscript. All calculations were checked and recalculated by CM to guarantee correctness. AD, AH and CM gave major suggestion on how to analyze and how to interpret the data and on the manuscript draft. All authors contributed to the design of the study and data collection. They also reviewed and edited the manuscript. All authors approved the final version of the manuscript.

## Pre-publication history

The pre-publication history for this paper can be accessed here:

http://www.biomedcentral.com/1471-2318/14/29/prepub
